# Persistence of Ambigrammatic Narnaviruses Requires Translation of the Reverse Open Reading Frame

**DOI:** 10.1128/JVI.00109-21

**Published:** 2021-06-10

**Authors:** Hanna Retallack, Katerina D. Popova, Matthew T. Laurie, Sara Sunshine, Joseph L. DeRisi

**Affiliations:** a Department of Biochemistry and Biophysics, University of California, San Francisco, California, USA; b Department of Cellular and Molecular Pharmacology, University of California, San Francisco, California, USA; c Chan Zuckerberg Biohub, San Francisco, California, USA; University of Kentucky College of Medicine

**Keywords:** CxNV1, ambigrammatic, narnavirus, reverse ORF, ribosome profiling

## Abstract

Narnaviruses are RNA viruses detected in diverse fungi, plants, protists, arthropods, and nematodes. Though initially described as simple single-gene nonsegmented viruses encoding RNA-dependent RNA polymerase (RdRp), a subset of narnaviruses referred to as “ambigrammatic” harbor a unique genomic configuration consisting of overlapping open reading frames (ORFs) encoded on opposite strands. Phylogenetic analysis supports selection to maintain this unusual genome organization, but functional investigations are lacking. Here, we establish the mosquito-infecting Culex narnavirus 1 (CxNV1) as a model to investigate the functional role of overlapping ORFs in narnavirus replication. In CxNV1, a reverse ORF without homology to known proteins covers nearly the entire 3.2-kb segment encoding the RdRp. Additionally, two opposing and nearly completely overlapping novel ORFs are found on the second putative CxNV1 segment, the 0.8-kb “Robin” RNA. We developed a system to launch CxNV1 in a naive mosquito cell line and then showed that functional RdRp is required for persistence of both segments, and an intact reverse ORF is required on the RdRp segment for persistence. Mass spectrometry of persistently CxNV1-infected cells provided evidence for translation of this reverse ORF. Finally, ribosome profiling yielded a striking pattern of footprints for all four CxNV1 RNA strands that was distinct from actively translating ribosomes on host mRNA or coinfecting RNA viruses. Taken together, these data raise the possibility that the process of translation itself is important for persistence of ambigrammatic narnaviruses, potentially by protecting viral RNA with ribosomes, thus suggesting a heretofore undescribed viral tactic for replication and transmission.

**IMPORTANCE** Fundamental to our understanding of RNA viruses is a description of which strand(s) of RNA are transmitted as the viral genome relative to which encode the viral proteins. Ambigrammatic narnaviruses break the mold. These viruses, found broadly in fungi, plants, and insects, have the unique feature of two overlapping genes encoded on opposite strands, comprising nearly the full length of the viral genome. Such extensive overlap is not seen in other RNA viruses and comes at the cost of reduced evolutionary flexibility in the sequence. The present study is motivated by investigating the benefits which balance that cost. We show for the first time a functional requirement for the ambigrammatic genome configuration in Culex narnavirus 1, which suggests a model for how translation of both strands might benefit this virus. Our work highlights a new blueprint for viral persistence, distinct from strategies defined by canonical definitions of the coding strand.

## INTRODUCTION

Narnaviruses are RNA viruses found broadly in eukaryotic hosts, including fungi, plants, protists, and arthropods (1). The narnavirus RNA-dependent RNA polymerase (RdRp) is most closely related to mitoviruses and ourmiaviruses and more distantly to the bacteriophage leviviruses, all of which are positive-sense single-stranded RNA viruses (+ssRNA) (2). The canonical narnavirus genome is simple—a single-stranded RNA 2.3 to 3.6 kb in length, encoding an RdRp (2). Double-stranded RNA (dsRNA) forms can be isolated, although these may represent by-products of RNA extraction, bona fide replication intermediates, translation templates, transmissible units, or some combination of the above (3, 4). Recently, putative second segments have been identified for two narnaviruses with likely protist hosts and for the arthropod-infecting Culex narnavirus 1 (CxNV1) and Zhejiang mosquito virus 3 (5–8). The function of proteins encoded by these segments is unknown, and their association with the RdRp segment is thus far only correlative. In many cases, narnavirus infection does not produce an observable phenotype and persists nonpathogenically, though examples exist where the narnavirus alters its host’s biology (9). From biochemical studies of narnaviruses in Saccharomyces cerevisiae and in nematodes, transmission is thought to occur via cytoplasmic inheritance during horizontal transfer in yeast or cell division, including animal germ lines, without any extracellular virion (10, 11). As described so far, narnaviruses would appear to exhibit a straightforward replication cycle. However, additional complexity in the genome organization of some narnaviruses may provide insight into a unique host-virus relationship that has not yet been described for any virus.

A subset of narnavirus genomes have a surprisingly complex feature consisting of an additional uninterrupted open reading frame (ORF) found in the reverse direction overlapping the RdRp ORF and spanning almost the entire length of the segment. Such narnaviruses have hence been described as “ambigrammatic,” as both the forward and reverse ORFs have the potential to be translated. This is achieved by avoidance of the codons UUA, CUA, and UCA, which encode stops in the −0 frame (1, 12). The extent of overlap in ambigrammatic narnaviruses is unprecedented in the catalog of known viral configurations.

Overlapping ORFs are common among viruses. Advantages include compact genomes, novel genes, and translation regulation, at the expense of evolutionary constraints imposed on the sequence by coding in two frames instead of one (13). Ambigrammatic narnaviruses are distinct from other RNA viruses because the ORF overlap is both extensive (>3 kb, comprising >95% of the genome) and antiparallel, pairing forward and reverse frames (1, 14, 15). Although ambigrammatic narnaviruses are diverse, with just 27% mean pairwise amino acid identity in their RdRps, the presence of a reverse ORF (rORF) is conserved in an apparently all-or-none fashion (1) and suggests a potential selection to retain the rORF. To our knowledge, the role of the ambigrammatic feature has not been investigated experimentally, and its function is wholly unknown.

These compelling observations motivate our investigation of CxNV1, an ambigrammatic narnavirus found in wild-caught mosquitoes. Its 3.2-kb RdRp-encoding RNA is associated with a second RNA, the 0.8-kb “Robin” segment. Both segments are ambigrammatic. Here, we analyzed the CxNV1 genome in a persistently infected cell line, developed a launching system to test the requirement for the rORF, examined rORF translation by mass spectrometry, and interrogated the association of ribosomes with each viral RNA by ribosome profiling.

## RESULTS

**FIG 1:**
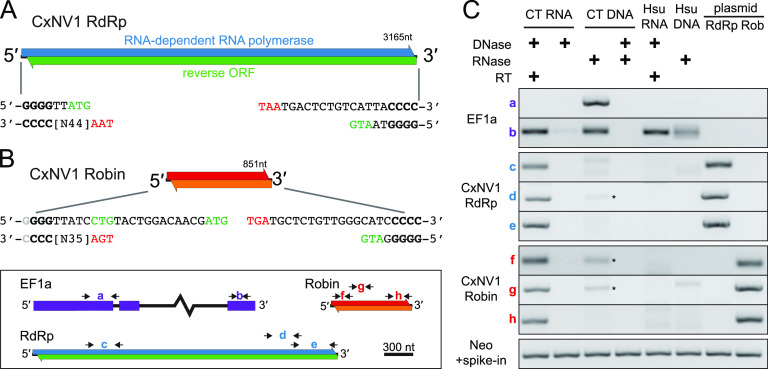
Characterization of CxNV1 in CT cell line. (A and B) Schematic of forward and reverse ORFs on the larger segment encoding the RdRp (A) and on the smaller segment, Robin (B). Consensus end sequences shown, with start codons in green and stop codons in red. The light gray nucleotide at the 5′ end of Robin indicates similar frequencies observed for 5′-GGGG and 5′-GGG. (C) RT-PCR on RNA and DNA from CT and Hsu cells, or on plasmids containing full-length clones of CxNV1 RdRp or Robin, treated with nucleases as indicated, with or without reverse transcriptase (RT) in the reaction. The key for primer pair locations is shown in the bottom left of the figure. The intronic primer specific to *C. tarsalis* in EF1a (primer pair a) indicates DNA recovery (absent from *C. quinquefasciatus* Hsu cells due to intronic sequence variation). Asterisks indicate faint bands at expected size in CT DNA. For reactions in the lowest row, a plasmid containing the neomycin (Neo) gene was spiked in and amplified using Neo-specific primers to verify that PCR amplification was not impeded by prior DNase treatment of CT/Hsu input.

To refine the genome of a model ambigrammatic narnavirus, we first characterized the complete genome sequence of CxNV1 found in the CT cell line derived from embryos of the Culex tarsalis mosquito (Fig. 1, Table S1 [supplemental files are available at https://doi.org/10.7272/Q6GX48SV]). Meta-transcriptomic next-generation sequencing (mNGS) revealed no evidence for other cellular organisms in the cell line, confirming *Culex tarsalis* as the host species (Fig. S1). An end-specific adapter-ligation sequencing approach revealed complementary ends on each CxNV1 segment, beginning with 5′-GGGG and ending with CCCC-3′ with a 21 nucleotide (nt) 3′ hairpin (Fig. 1A and B, Fig. S2, Tables S2 and S3). These features are conserved among narnaviruses (16). Overlapping ORFs on opposite strands extend nearly the full length of each segment. To further investigate similarities between the segments, the antiviral host cell response was assessed by reanalysis of small RNA sequencing data derived from the same CT cell line, as published by Göertz et al. (17). Abundant small RNAs aligning to both strands of the previously undetected CxNV1 Robin segment were present with a length mode of 21 nt, indicative of a small interfering RNA (siRNA) response similar to that against the RdRp segment (Fig. S3). The copy number ratio of Robin to RdRp segments was calculated at ∼3.8, based on length-normalized read abundance in mNGS libraries.

The unusual layout of ORFs raised the possibility of alternate RNA forms such as circular, subgenomic, or multiple defective viral RNAs. To address this question, we carefully examined the coverage profile of mNGS data and verified continuity with Sanger sequencing of >1-kb PCR products along the genome. For CxNV1, no evidence for alternate RNA forms was found. In contrast, for Calbertado virus (CALV), another persistent coinfection in CT cells, mNGS clearly showed a large increase in the coverage of the 3′ untranslated region (UTR) (Fig. S4), consistent with known subgenomic flaviviral RNAs (18). In summary, the RNA forms of both the RdRp and Robin segments of CxNV1 exhibit overlapping ORFs and complementary ends, are full-length linear RNAs, and are targeted by similar antiviral responses.

Next, we sought to determine whether DNA forms exist for CxNV1, which belongs to a family classified as +ssRNA viruses but infects insect cells which commonly endogenize viral sequences (19). As expected, reverse transcription-PCR (RT-PCR) yielded facile amplification of multiple products derived from both CxNV1 RdRp and Robin segments (Fig. 1C, Fig. S5). In contrast, amplification of CxNV1 from RNase-treated DNA was very inefficient, whereas amplification of the host EF1a genomic locus was robust, as expected. In all cases, Sanger sequencing of amplification products confirmed on-target product. Variants in the RNA mNGS data were then analyzed. Host genes such as EF1a and GAPDH contained single nucleotide variants (SNVs) at frequencies ranging from 0.45 to 0.50 which were phased as expected for two alleles derived from a diploid genome. In contrast, the SNVs observed in CxNV1 had frequencies far outside the bi-allelic range (<0.05 to 0.32), included sites with >2 variants, and were not phased (Table S4). Finally, the relative abundance of positive and negative strands was calculated and compared to characteristic ratios for host transcripts or viral transcripts from +ssRNA, dsRNA, or –ssRNA viruses. The relative abundance of positive to negative strand was ∼70 for the RdRp segment and ∼145 for the Robin segment, similar to ratios for CxNV1 found in wild-caught mosquitoes (7) and in the range of other +ssRNA and putative dsRNA viruses (Fig. S6).

Overall, these analyses support the bi-segmented nature of CxNV1 as an RNA virus with substantial negative-strand contribution. Importantly, the complete and accurate genomes allowed us to attempt to launch the virus in uninfected cells.

### Requirement of the reverse ORF.

**FIG 2:**
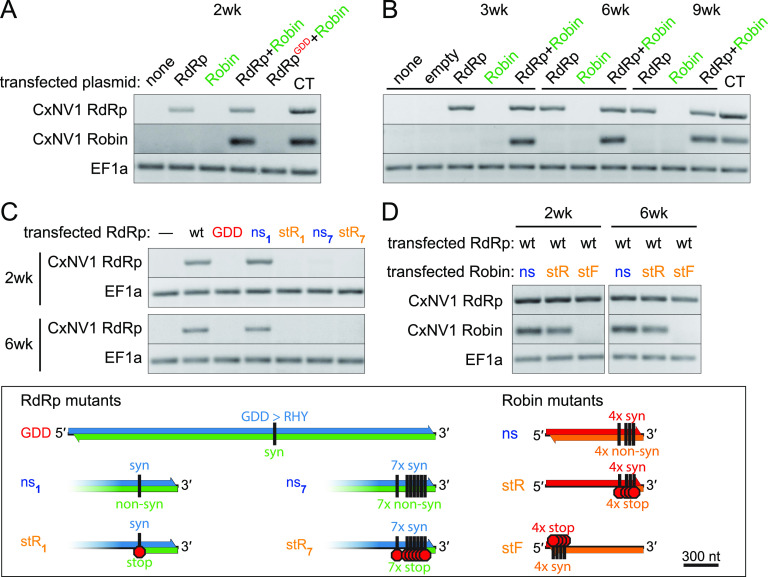
CxNV1 persistence depends on the GDD domain and reverse ORF in RdRp. (A) RT-PCR targeting CxNV1 RdRp, Robin, or EF1a RNA at 2 weeks posttransfection of Hsu cells with indicated plasmid and sort/countersort (see Materials and Methods) or CT cells as positive control. Plasmids drive expression of full-length positive-strand viral segments, either wild-type RdRp, inactive mutant RdRp (GDD), and/or wild-type Robin. (B) RT-PCR as in panel A for cells collected at 3, 6, and 9 weeks posttransfection. (C and D) RT-PCR as in panel A at 2 and 6 weeks posttransfection of Hsu cells with indicated plasmids, wild type (wt) or mutants diagrammed in key below. Key: Mutant RdRp constructs: mutations eliminating conserved motif required for polymerase activity (GDD); mutations introducing stop codons in the reverse ORF while remaining synonymous in the forward ORF (stR); mutations introducing nonsynonymous changes in the reverse ORF while remaining synonymous in the forward ORF all at the same nucleotide positions as stR (ns). Subscript (1 or 7) indicates number of codons mutated. Mutant Robin constructs: ns and stR mutants as for RdRp; mutations introducing stop codons beginning at 14 codons from the predicted start site of the forward ORF while remaining synonymous in the reverse ORF (stF). All experiments were performed in biological triplicate, with representative results shown here.

We developed a plasmid-based launch system to introduce CxNV1 into naive cells in order to test the functional requirement for each segment and their rORFs. Plasmids expressing full-length positive-sense CxNV1 were transfected into the narnavirus-free Hsu cell line derived from adult ovaries of the Culex quinquefasciatus mosquito (Fig. 2). After allowing plasmid loss through cell division, cells were counterselected using a fluorescence-activated cell sorter (FACS) to minimize expression from residual DNA. Although some lingering DNA was detectable, RT-PCR showed that the RdRp segment robustly persisted as RNA at least as far as 9 weeks posttransfection with regular cell passaging (Fig. 2A and B, Fig. S7 and S8). The Robin segment only persisted when cotransfected with RdRp, and both segments were undetectable when the GDD domain in the RdRp was mutated to render the polymerase inactive (Fig. 2A and B). These results demonstrate the operation of a CxNV1 launch platform which enables manipulation of the viral sequences.

Using this launch system for CxNV1 infection in cell culture, we next dissected the requirement for intact rORFs in the CxNV1 life cycle. First, mutations were introduced to interfere with the rORF while only introducing synonymous mutations on the opposing strand (Table S5). While a single nonsynonymous mutation in the rORF opposing RdRp had no effect (“ns1”), the introduction of a single stop codon in the rORF at the same position (“stR1”) resulted in loss of the RNA by 2 weeks posttransfection (Fig. 2C). Likewise, a more dramatic change introducing seven stop codons in the rORF (“stR7”) also resulted in loss of the RNA. Interestingly, introduction of seven nonsynonymous mutations into the rORF opposing RdRp also resulted in loss (“ns7”), suggesting that specific sequence elements in the 3′ region of the RdRp segment are important. Comparable mutations in Robin did not result in loss of the RNA in this assay (Fig. 2D). However, the Robin RNA was lost by 2 weeks when stop mutations were introduced near the beginning of the forward ORF, suggesting either required RNA *cis-*acting features or that persistence of the Robin RNA requires the protein encoded by its forward ORF. Targeted sequencing of the recovered RNAs showed that all mutations were retained without reversion to wild type. Together, these data provide evidence that a functional RdRp protein is required for replication of its own RNA segment as well as the Robin RNA segment and that an uninterrupted rORF is critical for CxNV1 RdRp persistence in this system.

### Interaction of ribosomes with CxNV1 RNA.

**FIG 3:**
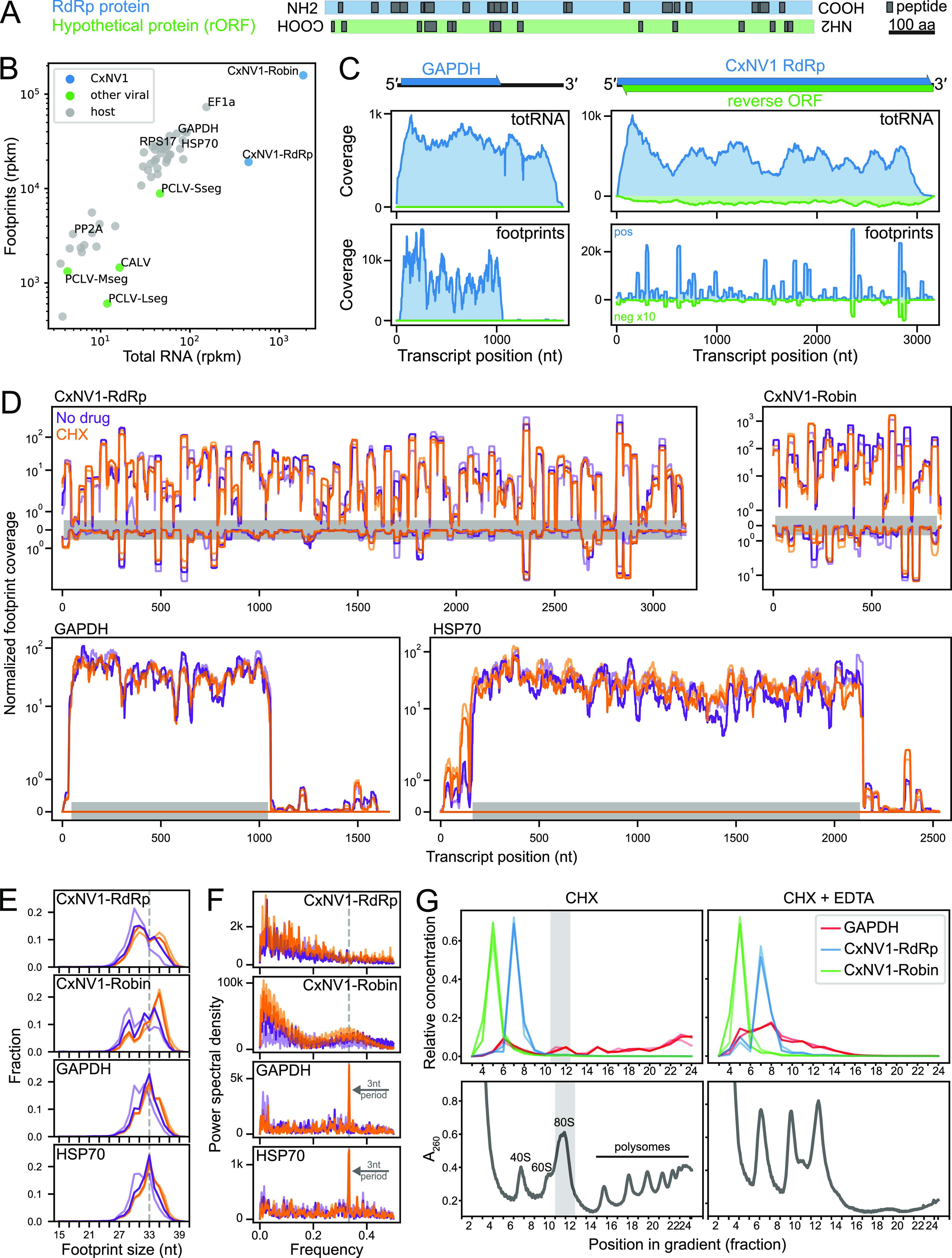
Investigation of ribosome interaction with each CxNV1 RNA strand and segment. (A) Alignment of peptides (in black) found by liquid chromatography tandem mass spectrometry (LC-MS/MS) of CT cell lysates to the amino acid sequence for the predicted RdRp and hypothetical proteins encoded by the forward and reverse ORFs, respectively, of the CxNV1 RdRp RNA segment. Displayed according to ORF layout in the genome. (B) Relative abundance of selected host transcripts and persistent viruses including CxNV1 in ribosome profiling (footprints) versus total RNA sequencing libraries. Data shown for positive strand, within-CDS densities, CHX-rep1 sample. (C) Read coverage of GAPDH and CxNV1-RdRp for ribosome profiling (footprints) and total RNA sequencing libraries. Reads mapping to negative strand in green, shown with 10-fold *y* axis magnification compared to positive-strand reads in blue for visualization purposes. Data shown for CHX-rep1 sample. (D) Ribosome profiling read coverage of each CxNV1 segment and two host transcripts. Data shown for two replicates of each condition, no drug (purple) and CHX (orange), using normalized values and displayed on a log_10_ scale. Gray bar indicates CDS position on the positive strand. Reads mapping to the negative strand are shown inverted. (E) Length distribution of footprints for each CxNV1 segment and two host transcripts in the ribosome profiling sequencing library. Colors indicate condition as in panel D. (F) Analysis of periodicity in footprints for each CxNV1 segment and two host transcripts. Ribosome densities from 5′ mapping were analyzed using Welch’s method to estimate the power spectral density at each frequency. A frequency of ∼0.33 (gray dashed line) corresponds to a period of 3 nt. Colors indicate condition as in panel D. (G) Polysome profiling of CT cells treated with CHX (left) or CHX+EDTA (right). Absorbance quantifying total RNA during fractionation of a single gradient for each condition is shown in lower panels. Relative concentrations of CxNV1-RdRp, CxNV1-Robin, and GAPDH RNA determined by RT-qPCR in upper panel, with replicates of independent gradients (*n* = 3 for CHX, *n* = 2 for CHX-EDTA). Gray shading indicates monosome peak.

The requirement for an intact rORF in the essential RdRp segment implies translation of the full-length rORF or other interaction with ribosomes. Mass spectrometry was used to investigate translation in persistent CxNV1 infection. Peptides were detected from across the entire length of both forward and reverse ORFs on the RdRp segment (Fig. 3A, Table S6), confirming that translation occurs on both strands of the RdRp segment.

Next, ribosome profiling was performed to explore the translational landscape of CxNV1 RNAs. Ribosome profiling libraries were prepared in biological duplicate from the micrococcal nuclease (MNase)-digested monosome fraction of CT cells with either cycloheximide (CHX) or no treatment (no-drug) before lysis (Table S7). RNAseq libraries were prepared from total RNA (totRNA) of the same samples using an mNGS workflow. As the additional pretreatment had little effect, the four libraries were considered near replicates.

Forty-eight host transcripts were *de novo* assembled for the following analyses, as no genome is currently available for *Culex tarsalis* (Table S8; see Materials and Methods). Among these host transcripts, a consistent ratio of footprint to total RNA reads was observed (Fig. 3B, Fig. S9), with footprint reads aligning within the coding sequences (CDS) on the positive-sense RNA as expected (Fig. 3C and D, Fig. S10).

For CxNV1, footprints were observed on both strands, at a strand ratio similar to that observed in the total RNA libraries (Fig. S11). However, footprints on CxNV1 showed several unexpected features. First, the abundance of footprints relative to total RNA abundance was substantially lower for CxNV1 than for host transcripts (Fig. 3B, Fig. S9). Second, the footprints were heavily concentrated at specific positions, resulting in an unusual profile (Fig. 3C). This “plateau pattern” in coverage was observed on both CxNV1 segments, in all four libraries, in contrast to the more uniform coverage of typical footprint distributions along host genes such as GAPDH and HSP70 (Fig. 3D, Fig. S12). Third, the lengths of footprints mapping to CxNV1 were broadly distributed from 27 to 37 nt and not enriched in the 33-nt size that dominated host genes (Fig. 3E, Fig. S13). Fourth, triplet periodicity, which is expected for normally translating mRNAs, was not observed for CxNV1. For most host transcripts, Fourier transform analysis revealed greatest power at a frequency of 0.33 (period of 3 nt), and the nucleotide composition at footprint-adjacent positions was consistent with codon-based ribosome positioning (Fig. 3F, Fig. S14 and S15A). The imprecision of MNase digestion prevented highly accurate read phasing. The possibility of weak periodicity due to MNase sequence specificity rather than ribosome reading frame was examined further below (20). Taken together, these differences in CxNV1 ribosome profiling point to a potentially distinct type of interaction between both strands of this virus and the host translation machinery.

We performed a series of additional analyses to determine whether technical factors could explain these observations. With regard to sequencing, insufficient sampling as a source for CxNV1-specific footprint patterns was ruled out, as an ample number of footprint reads were observed for both CxNV1 segments, and low-abundance host transcripts such as PP2A did not show the plateau pattern. PCR jackpotting was also considered a potential confounder. Removing PCR duplicates by collapsing identical footprint + unique molecular identifier (UMI) sequences caused greater depletion of CxNV1-mapping footprints than other genes (Fig. S16 and S17). However, the extreme concentration of CxNV1 footprint peaks could exceed the diversity in a random 5-nt UMI library. Regardless, the CxNV1-specific plateau pattern was retained after duplicate collapse.

Next, steps upstream of sequencing were examined. Sequence biases inherent to multiple steps in sample preparation, including nuclease digestion, adapter ligation, and circularization, are known to affect footprint recovery (21, 22). For instance, MNase is known to cleave more efficiently 5′ of A and T than of C or G (23), a bias also observed for all transcripts in our data (Fig. S15B). To ascertain whether this nucleotide bias was sufficient to account for the CxNV1-specific pattern, a model was developed for footprint distribution based on empirical nucleotide frequencies at the footprint edge and footprint length. No significant difference was seen in the correlation between the observed coverage on CxNV1-RdRp and the model-predicted footprint coverage using either the actual CxNV1-RdRp sequence or a scrambled sequence (Fig. S18), suggesting that nucleotide bias at MNase cut sites alone is unlikely to yield the plateau pattern in CxNV1.

We examined whether the unusual CxNV1 footprints could be attributable to abundant viral RNA or antiviral pathways, RNA secondary structure, or features at the amino acid level. Footprint data for CxNV1 were first compared to other persistent viruses in the cell line, including CALV and the negative-sense, tri-segmented Phasi Charoen-like phasivirus (PCLV). For CALV and PCLV, relatively uniform footprint coverage was observed across the canonical ORFs with expected drop-offs in the 3′ UTRs (Fig. S10). To assess whether the CxNV1-specific footprints represent nonspecific interactions with abundant RNAs, footprints on the genomic negative strand of PCLV were examined, as an example of an abundant RNA that is likely not translated. The negative-strand PCLV footprints were less abundant than positive-strand footprints (Fig. S11), did not show the CxNV1-specific plateau pattern (Fig. S10), and had length distributions that were significantly different from the reference EF1a positive-strand distribution (PCLV Lseg and Mseg, Fig. S13), suggesting that the negative-strand reads for PCLV represented a different process than CxNV1 footprints. Noncoding host transcripts for additional comparison could not be confidently identified given the lack of existing genome for this species. Lastly, the distribution of footprints in our ribosome profiling was related to mapping of small RNAs previously sequenced from the same cell line by comparing coverage profiles. No direct correlation or consistent pattern was observed for CxNV1 (Pearson correlation coefficient [*r*] and *P* value for RdRp, *r *= 0.02, *P* = 2.0e-1; Robin, *r*=−0.14, *P* = 2.8e-5). In summary, the plateau pattern of CxNV1 footprints was not solely attributable to abundant viral RNA or antiviral pathways.

We next focused on RNA secondary structure, which is often critical for ribosome recruitment by viruses lacking a 5′ cap and other interactions with nonribosomal RNA-binding proteins (RBPs) (24, 25). To approximate the local RNA structure, the mean free energy (MFE) of predicted folding was calculated using sliding windows along each transcript (see Materials and Methods). The resulting secondary structure profile did not reveal large internal hairpins in CxNV1 and did not correlate with the footprint profile nonribosomal RNA-binding proteins (RBPs) (Fig. S19). With aligned footprint positions, small local peaks of MFE were observed at 5′ and 3′ footprint edges in CxNV1. This modest trend was more apparent in CxNV1 than host genes and was supported in only some regions by mapping footprint densities onto RNA structures predicted from 150- to 200-nt stretches. Overall, no strong evidence was found to support internal RNA secondary structure as a contributing nonribosomal RNA-binding proteins (RBPs) factor to the CxNV1-specific footprint profiles.

Amino acid features were also considered. In yeast, codons encoded by less abundant tRNAs tend to be translated more slowly (26). Due to the imprecision of MNase cutting and the absence of an annotated genome for our data set, A/P/E-sites could not be confidently assigned, and codon-based analyses were not performed. It is also possible that features of the nascent polypeptide chain may affect translation elongation and result in footprint pileup, such as the presence of poly-proline stretches or the charge of ∼20 to 30 residues passing through the eukaryotic ribosome exit tunnel (27–31). No significant bias in charge, hydrophobicity, or individual amino acids enriched in the 20 residues upstream of approximate P-site positions was found (Fig. S20).

Finally, polysome profiling was performed to ascertain ribosome density on CxNV1 RNA. Reverse transcription-quantitative PCR (RT-qPCR) targeting CxNV1 and GAPDH was performed on fractions from the polysome gradient of undigested RNA, with or without EDTA treatment of lysates before gradient separation to release RNA-bound complexes including ribosomes (Fig. 3G, Fig. S21). GAPDH RNA in untreated lysates was found in fractions corresponding to monosomes and polysomes and in lighter fractions after EDTA treatment. Conversely, the vast majority of CxNV1 RNA was found in fractions lighter than monosomes regardless of EDTA treatment and thus would not be expected to contribute to footprinting data. Furthermore, these data are not consistent with the idea that the CxNV1 profile represents a summation of many sparsely footprinted RNAs. Instead, they raise the possibility that the observed footprints are derived from a small amount of heavily protected RNA.

## DISCUSSION

Overlapping genes are important features of many viruses, with functions ranging from novel gene creation to regulation of expression. Among RNA viruses without DNA intermediates, the vast majority of ORF overlaps are <1.5 kb and occur in the same direction (13, 14, 32), and translation of overlapping antisense ORFs has never been experimentally demonstrated (1, 15). Thus, the antisense 3.1-kb overlap in CxNV1 is especially striking. Investigation of this ambigrammatic narnavirus has been previously limited to *in silico* analyses. Here, we leveraged a naturally persistent infection by CxNV1 of CT cells to address unique aspects of CxNV1 biology, including the relationship between the two segments, the requirement for the rORF, and the translational landscape of this unconventional virus.

Our data suggest that CxNV1 persists predominantly as RNA in CT cells. While the amount of dsRNA was not explicitly quantified in this study, the overabundance of positive-strand RNA implies that the possible dsRNA fraction may be no more than 1 to 2% of the total, if it exists. This fraction is also an upper bound on the proportion of viral RNAs actively involved in replication at any given moment. The faint signal of an RNA virus detected in the DNA fraction of infected CT cells is not unprecedented (33). While viral sequences are frequently endogenized in insect cells (19, 34, 35), a bi-allelic pattern of SNV frequency expected for such endogenized sequences was not observed for CxNV1, and thus the data are more consistent with incidental reverse transcription of the CxNV1 RNA.

The presence of a second segment associated with CxNV1 (the Robin segment) is a relatively new and unexplored finding. Our data suggest that CxNV1 RdRp is required for replication of the Robin RNA. While the RdRp segment persisted without Robin in cell culture, this RdRp-only state has not been observed for CxNV1 in wild-caught mosquitoes (7). It is possible that Robin provides a fitness benefit that is only apparent in the context of the complete organism or on a time scale not reproduced by cell culture. Robin may also have a niche role for CxNV1-like or ambigrammatic narnaviruses specifically, as the CxNV1 Robin genome organization is unlike those of non-RdRp segments identified in the distantly related nonambigrammatic narnaviruses LepseyNLV1, MaRNAV-1, and MaRNAV-2, (5, 6). A putative second segment for Zhejiang mosquito virus 3, also ambigrammatic, was computationally identified during revision of this manuscript (8). Additional sequences in the phylogenetic tree are needed to clarify the evolutionary history of the Robin segment and whether its relationship to the RdRp is an example of viral parasitism or symbiosis.

The relationship between the RdRp and Robin segments of CxNV1 may hinge on the shared feature of overlapping ORFs on opposite strands. Our experiments demonstrated that premature truncation of the rORF on the RdRp segment resulted in loss of the RNA, indicating a requirement for the presence of the rORF. As for the importance of the rORF protein sequence, we note that the forward ORFs are considerably more conserved than the rORFs, as shown in a previously published data set of related CxNV1 sequences (7), where the number of variable positions per 100 amino acids decreases as follows: 47 in the Robin rORF > 40 in the RdRp rORF > 36 in the Robin forward ORF > 13 in the RdRp forward ORF encoding the RdRp protein. The lack of rORF sequence conservation may indicate that the act of translation is important, rather than a specific protein product.

**FIG 4:**
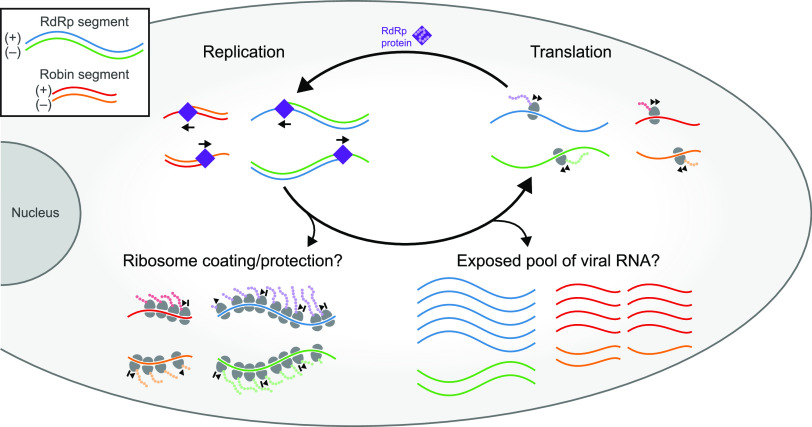
Model of CxNV1 infection. The RdRp segment encodes the viral RNA-dependent RNA polymerase (RdRp), which replicates both the RdRp and Robin segments to generate (+) and (–) strand viral RNA. A small fraction of the total viral RNA may be densely covered in ribosomes. These ribosomes may queue in predictable locations behind paused ribosomes, thus protecting the viral RNA. The remaining pool of viral RNA may be exposed to nucleases. Actively translating ribosomes also produce proteins from the ORFs on each strand of each segment. The role of proteins encoded by the three non-RdRp ORFs is unknown. Ultimately, unconventional interaction of ribosomes with each strand may be more important for the persistence of ambigrammatic narnaviruses than the protein products of translation.

What, then, is the role of the essential overlapping opposite-sense ORFs? While the rORF appears to be translated, the bulk of viral RNAs appear to be devoid of ribosomes. Assuming that the footprints observed in the ribosome profiling libraries truly indicate ribosome-protected RNA, then the interaction of ribosomes with CxNV1 is unlike typical host RNA translation. We propose a model where a small fraction of CxNV1 RNA is occupied by regions of densely packed ribosomes (Fig. 4). This configuration, on each strand of both segments, might result from one or more stall points with ribosome queueing, yielding discrete positions with 30- to 40-nt spacing. It has long been known that stacking of up to 10 ribosomes can occur (36), and recent attention on disomes suggests that ribosome collisions may be a widespread phenomenon on eukaryotic cellular transcripts (37–39), prompting the question of how CxNV1 might interact with mechanisms for handling ribosome collisions (40). The possible causes of ribosome pausing, such as cryptic RNA secondary structure, are unclear. Likewise, the mechanism by which CxNV1 regulates competition between replication and translation is unknown, and our data suggest that a substantial portion of the viral RNA is neither actively involved in replication nor bound by ribosomes.

We can only speculate on how ribosome-coating of viral RNAs might contribute to viral fitness. Perhaps ribosomes fill roles typically performed by nucleocapsid proteins in other viruses, such as protecting the viral RNA from host cell degradation, maintaining RNA in a polymerase-accessible state, or influencing transmission of viral RNA during cell division. Further study of CxNV1 RNA stability in the context of various deficiencies in host cell antiviral pathways may be informative.

Alternative interpretations of the data, including technical caveats, were considered, but these were found to be insufficient to explain the observations. We considered and rejected artifacts from PCR and library preparation steps and bias in nucleotide and amino acid sequence. Conceivably, the footprints might represent contaminating RNA or protection by a nonribosomal RBP found at the same sucrose density as the monosome peak. The unusual footprint size distribution for CxNV1-mapping footprints warrants consideration. On one hand, nonconforming size distributions are often used to exclude regions lacking bona fide translation activity, as in many noncoding RNAs and 3′ UTRs (41). For unusual-sized footprints mapping to noncoding strands of RNA viruses, including influenza and coronaviruses, some groups have hypothesized that the footprints reflect protection by viral nucleoprotein complexes (of note, these protocols isolate footprints using size exclusion chromatography spin columns or sucrose cushion rather than purifying monosomes via sucrose gradient as was used here) (42–45). On the other hand, footprint size can vary according to ribosome conformation, which may in turn be determined by the stage of translation and/or stalled or collided state that is captured at the time of digestion (46). Our technical approach may have captured RNA protected by single ribosomes in unusual conformations. For CxNV1, nonconforming footprint sizes could be expected from ribosomes that are stacked or otherwise not translating efficiently. It is also possible that ribosomes on the viral RNA bear modifications or protein interactions distinct from translating complexes on host mRNAs that could affect footprint size. Although nonribosomal RBP footprints cannot be excluded, our model is more parsimonious in joining the observations of dual-ORF conservation and the footprinting data.

In conclusion, this investigation suggests a previously unappreciated strategy among viruses, whereby a small fraction of the RNA from this unique narnavirus exists in a state characterized by bound, densely packed, nonprocessive ribosomes. This may represent a novel mechanism by which an RNA virus can coopt the host translational machinery to facilitate persistent infection and transmission in the absence of an intact virion and extracellular phase.

## MATERIALS AND METHODS

### Cell culture.

Established cell lines derived from *Culex tarsalis* embryos (CT) and Culex quinquefasciatus ovaries (Hsu) were a generous gift of Aaron Brault (Centers for Disease Control and Prevention, Fort Collins, CO, USA) (47, 48). Cells were grown in Schneider’s *Drosophila* medium (Gibco) supplemented with 10% vol/vol fetal bovine serum at 28°C in room air, passaged by scraping, and tested negative for mycoplasma monthly.

### Virus NGS and PCR.

To sequence the parental CT cell line, RNA was first extracted using a Direct-Zol RNA MicroPrep kit (Zymo Research). After adding External RNA Control Consortium (ERCC) RNA spike-in mix (Life Technologies), the sequencing library was prepared using a NEBNext Ultra II directional RNA library prep kit (New England Biolabs [NEB]) and sequenced with a paired-end 150-bp run on an Illumina NextSeq instrument. The raw reads were processed via the IDSeq pipeline (v4.6; using the NCBI's nucleotide nonredundant [nt/nr] database from 2020-02-10) for initial processing, including quality filtering, deduplication, host subtraction, *de novo* assembly, and identification of viral sequences (49). These filtered reads and assembled contigs were used for analyses of the persistent viruses, including positive to negative-strand ratios, consensus viral sequences, and variant analyses. Bowtie2 (v2.2.4) was used for alignments to correct any assembly errors (50). Independently, raw reads were quality filtered via the PRICE sequence filter (v1.2, PriceSeqFilter) and used for host analyses, including building the host transcriptome reference as described in the “Ribosome Profiling Analysis” section below (51).

Two strategies were used to identify the ends of the CxNV1 RNA, ligation to RNA (for the 3′ end) and reverse transcription and then ligation to cDNA (for the 5′ end). See Table S9 for oligo sequences. For both strategies, the oligo ligated to the unknown end contained a handle TATGCA followed by 0 to 5 Ns before the Illumina TruSeq Adapter 5 (TSA5) sequence to allow for dephasing during read1 sequencing (oHR546/547/548/549/550/551). For the RNA ligation method, these ligation oligos were ordered with 5′ phosphorylation and 3′ blocking with C3 Spacer (IDT), pooled in equimolar ratios, preadenylated at the 5′ end using Mth RNA ligase (NEB), and then ligated to total RNA extracted from CT cells using 5′ App DNA/RNA ligase (NEB). Next, a TSA5-complementary oligo (oHR552) was used to initiate reverse transcription with SSIII (Thermo Fisher) followed by basic RNA hydrolysis and column purification (DNA Clean and Concentrator; Zymo Research). Then, the regions of interest were amplified and barcoded using nested PCR with Phusion high-fidelity DNA polymerase (NEB), an internal primer containing TSA7 and a footprint sequence ∼100- to 150 nt from the predicted end (oHR542/543/544/545, 0.1×), and external primers containing the P5/P7 and i5/i7 Illumina adapter and index sequences.

For the cDNA ligation method, targeted reverse transcription was performed on the extracted CT cell RNA with the footprint oligos oHR542/543/544/545 and SSIII (Thermo Fisher), which is known to add a few nontemplated bases after reaching the 5′ end of the RNA with a preference for cytosine (52). The pooled 3′-blocked, 5′-preadenylated oligos oHR546-551 were then ligated to the cDNA using 5′ App DNA/RNA ligase (NEB). Amplification and barcoding PCR were then performed with oligos that annealed to the TSA5 and TSA7 sequences and added i5/i7 and P5/P7 sequences. All reactions were performed according to the manufacturer’s instructions. The final amplicon libraries were size-selected using AMPure XP beads (Beckman Coulter) at 0.9× and then quantified using a Qubit dsDNA HS assay kit (Thermo Fisher) and size-verified with BioAnalyzer (Agilent). Libraries that failed to enrich for the expected peak size (likely due to nonspecific primer binding and inefficiency of ligating to total RNA before reverse transcription) were dropped at this stage. Final libraries were pooled and sequenced with a paired-end 150-bp run on an Illumina NextSeq instrument.

For analysis of ends libraries, reads were first quality filtered using PriceSeqFilter with the flags -rqf 85 0.98 -rnf 90 and then trimmed to the read beyond the handle TGCATA. Reads were then aligned to the consensus sequence for the region of CxNV1 well supported by >4 reads from mNGS sequencing (see above) using Bowtie2 (v2.2.4). Variants in the terminal 8 bases were analyzed to determine the most likely consensus sequence, taking into account potential biases from reverse transcription and ligation and potential biological variation. RNA folding of terminal sequences was performed using the RNAstructure web server with temperatures according to narnavirus host species: 28°C for insect, 30°C for yeast (53).

Experiments to determine whether CxNV1 has DNA forms were initiated by extracting RNA from CT cells using a Direct-Zol RNA MicroPrep kit (Zymo Research) and, separately, DNA from matched aliquots of CT cells using a Quick-DNA miniprep kit with proteinase K (Zymo Research). As a control, RNA and DNA were also extracted from Hsu cells. Extracted RNA was treated with DNase during column purification, and extracted DNA was treated with RNaseA (Thermo Fisher) and/or with DNase (Invitrogen) as a control. Amplification reactions were performed using KAPA SYBR FAST one-step qRT-PCR (Roche), with or without reverse transcriptase, on the purified RNA or DNA spiked with a Neo-containing plasmid, with 35 cycles, 60°C annealing temperature, 30 sec extension, and the following primer pairs: EF1a oHR561/565 (intronic) and oHR672/673 (exonic); CxNV1 RdRp oHR504/509, oHR502/503, and oHR513/514; CxNV1 Robin oHR653/654, oHR538/539, and oHR540/541; and Neo oHR691/692 (Table S9). PCRs and agarose gel electrophoresis to analyze their products were run in parallel for all conditions. To determine the identity of faint bands at similar size to target products, reactions were rerun with 40 cycles, and then bands were cloned using Topo TA cloning (Thermo Fisher) and Sanger sequenced.

### Reanalysis of published sequencing data.

Small RNA sequencing of CT cells with or without infection with West Nile virus (WNV) was previously performed by Göertz et al. (17). The data sets from the NCBI’s Sequence Read Archive (SRA) (accession numbers SRR8668667 and SRR8668668) were downloaded, adapter sequences were trimmed as needed using Trimmomatic (v0.39) with Illumina TruSeq smallRNA adapters and flags ILLUMINACLIP:$adapter_fasta:1:0:0:1 MINLEN:18, quality filtered using PriceSeqFilter with flags -rqf 85 0.98 -rnf 90, and then aligned to the combined CT host plus virus transcript reference (see “Ribosome Profiling Analysis” section) using Bowtie (v1.2.3) with flags -v 1 -k 1 -m 1.

mNGS of wild-caught mosquitoes in California was previously performed by Batson et al. (7). Quality-filtered reads from the data set available in the SRA (BioProject number PRJNA605178) were aligned to the assembled contigs for each sample for contigs assigned to viral species by the original study authors. The ratio of concordantly aligned read pairs derived from positive (coding) versus negative strand was calculated. Viral segments with <100 concordantly mapped read pairs or <3 samples (individual mosquitoes) were discarded from the analysis.

### Plasmids.

To clone CxNV1 from CT cell RNA, an iterative process of RT-PCR (Superscript III platinum 1-step RT-PCR; Thermo Fisher) and topo cloning (Invitrogen) of long genomic fragments was performed, ultimately completed with primers oHR594/593, oHR592/586, oHR585/589, and oHR619/620 for 4 fragments of RdRp and with oHR621/622 and oHR623/624 for 2 fragments of Robin, each flanked by BbsI or BsaI cut sites (Table S9). The fragments were assembled into a pUC19 vector using In-Fusion cloning (TaKaRa Bio) with inverse PCR to add ends determined by the ligation sequencing method described above, ultimately generating plasmids pHR96 (RdRp) and pHR97 (Robin), which contain SmaI-flanked full-length genomic RNAs.

Mutant versions of RdRp and Robin were generated with a combination of PCR, GeneBlocks (IDT), restriction enzyme, and In-Fusion cloning. To control for effects of mutations at the RNA level, mutants with stop codons were compared to mutants with nonsynonymous changes at identical nucleotide positions. Unique restriction enzyme sites were introduced to distinguish plasmids easily. Details can be found in Table S5.

To generate plasmids for expression of CxNV1 RNAs in insect cells, a backbone was first created by inserting the T7-HDR (hepatitis D ribozyme) region from p2RZ (gift from Kristeene Knopp) into pIEX-4 (gift from Wesley Wu) and then inserting this cassette into pAc5-STABLE1-Neo (gift from Rosa Barrio and James Sutherland; Addgene plasmid number 32425), generating “empty” plasmid pHR106. The RdRp or Robin segments were inserted into this backbone, generating pHR107 and pHR108, respectively, each containing separate Ac5-GFP and hr5/IE1-narnavirus expression constructs. In these plasmids, narnaviral RNA is transcribed with ∼72 nt upstream of the viral 5′ terminus and with the hepatitis D ribozyme to cleave at the viral 3′ terminus. In addition, the green fluorescent protein (GFP) was swapped for mCherry in expression plasmids containing CxNV1-Robin.

### Virus launch.

To launch CxNV1 in a naive cell line, Hsu cells were transfected using X-tremeGENE HP DNA transfection reagent (Roche) with plasmid(s) expressing a fluorophore under the control of the Ac5 promoter, and the CxNV1 RdRp, Robin, mutants, or empty under the control of the IE1 promoter with hr5 enhancer, as described above. In initial experiments, both segments were in GFP-expressing vectors; subsequent experiments were performed with Robin in a backbone expressing mCherry instead of GFP, showing that when multiple plasmids were transfected concurrently, >95% of cells received either both or neither plasmid, and yielding no difference in results. As antibiotic-based selection was inefficient, cells were first sorted for successful transfection at 2 to 6 days posttransfection (fluorophore-positive) and then passaged and countersorted for loss of plasmid at 10 to 12 days posttransfection (fluorophore-negative) using a Sony SH800S sorter. At time points from 2 to 9 weeks posttransfection, cells were collected for nucleic acid extraction (including DNase treatment for RNA extractions) and PCR amplification. To determine persistence of viral RNA after loss of plasmid DNA, RT-PCRs were performed with the following primer pairs: oHR672/673 (EF1a), oHR513/514 (CxNV1 RdRp), oHR653/654 (CxNV1 Robin), and oHR691/692 (Neo) (Table S9).

### Mass spectrometry.

Lysates were prepared from two 15-cm tissue culture plates of CT cells by lysing in ice-cold radioimmunoprecipitation assay (RIPA) buffer (10 mM Tris-HCl, pH 7.4, 1% vol/vol Triton X-100, 0.1% mass/vol SDS, 140 mM NaCl) with cOmplete EDTA-free protease inhibitor cocktail (Roche) and then rotating overhead for 10 min at 4°C, followed by centrifuging for 10 min at 16,000 × *g* at 4°C and flash-freezing the supernatant. The protein concentration was measured using the Bradford assay (Bio-Rad). Lysates were diluted in 2× sample buffer (4% mass/vol SDS, 20% vol/vol glycerol, 120 mM Tris-HCl, 0.02% mass/vol bromophenol blue) supplemented with 10% vol/vol beta-mercaptoethanol, boiled at 95°C for 3 min, sheared with a 26G needle, boiled 2 min, and centrifuged, and then 15 μg was loaded onto a NuPAGE 4 to 12% Bis-Tris polyacrylamide gel (Invitrogen) and separated by electrophoresis. Gel bands were cut from two regions, 100 to 150 kD and 25 to 37 kD, and processed via trypsin digestion and liquid chromatography tandem mass spectrometry (LC-MS/MS) as detailed below. Coomassie stain of parallel gels confirmed adequate separation of the complex lysate. Biological duplicates were performed 10 months apart.

Mass spectrometry was performed by the Vincent J. Coates Proteomics/Mass Spectrometry Laboratory at the University of California at Berkeley (UC Berkeley). A nano LC column was packed in a 100-μm-inner diameter glass capillary with an emitter tip. The column consisted of 10 cm of Polaris c18 5-μm packing material. The column was loaded by use of a pressure bomb and washed extensively with buffer A (see below). The column was then directly coupled to an electrospray ionization source mounted on a Thermo Fisher LTQ XL linear ion trap mass spectrometer. An Agilent 1200 high-pressure liquid chromatography (HPLC) system equipped with a split line so as to deliver a flow rate of 300 nL/min was used for chromatography. Peptides were eluted with a 90 minus gradient to 60% B. Buffer A was 5% acetonitrile/0.02% heptafluorobutyric acid (HBFA); buffer B was 80% acetonitrile/0.02% HBFA.

Protein identification was done with the Integrated Proteomics Pipeline (IP2; Integrated Proteomics Applications, Inc., San Diego, CA) using ProLuCID/Sequest, DTASelect2, and Census (54–57). Tandem mass spectra were extracted into ms1 and ms2 files from raw files using RawExtractor (58). Data were searched against a database consisting of the Culex quinquefasciatus and custom viral databases supplemented with sequences of common contaminants. The database was concatenated to a decoy database in which the sequence for each entry in the original database was reversed (59). LTQ data were searched with 3,000.0 milli-amu precursor tolerance, and the fragment ions were restricted to a 600.0 ppm tolerance. All searches were parallelized and searched on the Vincent J. Coates proteomics cluster. Search space included all fully tryptic peptide candidates with no missed cleavage restrictions. Carbamidomethylation (+57.02146) of cysteine was considered a static modification. Phosphorylation was searched as a variable modification. We required 1 peptide per protein and both trypitic termini for each peptide identification. The ProLuCID search results were assembled and filtered using the DTASelect program (54, 56) with a peptide false-discovery rate (FDR) of 0.001 for single peptides and a peptide FDR of 0.005 for additional peptides for the same protein.

### Ribosome profiling.

CT cells were pretreated with 100 μg/mL cycloheximide (CHX; Sigma) at 37°C for 2 min. Ribosome-protected footprints from cycloheximide-pretreated and no-drug samples were prepared for sequencing as described in a recently updated protocol for ribosome profiling of mammalian cells, with slight modifications (60). Briefly, cells were rapidly harvested at 4°C in lysis buffer (20 mM Tris-HCl, pH 7.4, 150 mM NaCl, 5 mM MgCl_2_, 1 mM dithiothreitol [DTT], and 100 μg/mL CHX, supplemented with 1% vol/vol Triton X-100 and 25 U/mL Turbo DNase I [Thermo Fisher]). Clarified cell lysates were treated with micrococcal nuclease (MNase) to digest RNA not protected by ribosomes. MNase has previously been effective for footprinting insect cells (61), and it has been suggested that RNase I may degrade insect ribosomes (62). In our hands, MNase digestion produced a similar increase in the monosome (80S) fraction in CT cells as RNase T1 (Thermo). 80S ribosomes were isolated by centrifuging lysates through a 10 to 50% mass/vol sucrose gradient at 35,000 rpm for 3 h at 4°C with an SW41 rotor on a Beckman L8-60M ultracentrifuge and then collecting the monosome fraction on a BioComp gradient station. RNA was then purified from the monosome fractions using a Direct-Zol RNA kit (Zymo Research) and then resolved by electrophoresis through a denaturing gel, and the fragments corresponding to ∼26 to 34 bp were extracted.

The 3′ ends of the ribosome footprint RNA fragments were then treated with T4 polynucleotide kinase (NEB) to allow ligation of a preadenylated DNA linker with T4 Rnl2(tr) K227Q (NEB). The DNA linker incorporates sample barcodes to enable library multiplexing, as well as unique molecular identifiers (UMIs) to enable removal of duplicated sequences. To separate ligated RNA fragments from unligated DNA linkers, 5′-deadenylase (Epicentre) was used to deadenylate the preadenylated linkers, which were then degraded by the 5′-3′ ssDNA exonuclease RecJ (NEB). After rRNA depletion using the Ribo-Zero Gold rRNA removal kit (Illumina), the RNA-DNA hybrid was used as a template for reverse transcription with Superscript III (Thermo), followed by circularization with CircLigase (Epicentre). Finally, PCR of the cDNA circles attached suitable adapters and indices for Illumina sequencing and then libraries were quantified and pooled. The library was sequenced with a single-end 50-bp run on an Illumina HiSeq 4000 instrument.

The corresponding RNA-seq samples were prepared from total RNA of the same cell lysates. RNA was extracted using a Direct-Zol RNA MicroPrep kit (Zymo Research), and then libraries were prepared using a NEBNext Ultra II directional RNA library prep kit (NEB) and, finally, sequenced with a paired-end 150-bp run on an Illumina NextSeq instrument.

### Ribosome profiling analysis.

No genome reference was available for *Culex tarsalis*. The closest species, Culex quinquefasciatus, is homologous enough in exonic regions for credible alignments of 150-nt paired-end reads, but we found noncoding regions including introns to be dissimilar enough to make genome-wide alignments unreliable and annotations somewhat lacking. Instead, we chose to assemble a set of transcripts likely to be well conserved and spanning from low to high abundance, namely, genes for elongation factors, initiation factors, and ribosomal proteins, and a few likely single-copy canonical genes, EF1a, GAPDH, HSP70, RPS17, and PP2A. We abandoned genes where the annotations were unclear and which may have multiple copies in the genome. We first extracted mRNA sequences for genes of the classes listed above from the CulPip1.0 genome assembly tool (NCBI). We then aligned the quality-filtered reads from the 150-nt paired-end sequencing of the parental CT cell line. For transcripts with promising homology, we extended the contig into 5′ and 3′ UTRs using PriceTI (v1.2) and then validated by remapping paired-end reads and, finally, selecting transcripts with unambiguous mapping at >4 read coverage. See Table S8 for BLAST results of final host reference transcripts.

The genomes for persistent RNA viruses in the CT cell line were generated by aligning 150-nt paired-end reads to the contigs assembled with SPAdes in the IDSeq pipeline for error correction and contig extension. Culex narnavirus 1 (CxNV1), Calbertado virus (CALV), and Phasi Charoen-like phasivirus (PCLV) were assembled this way. Anomalies in the alignments and assembly of Flock House virus (FHV) were suspicious for defective viruses or other multiplicity, so FHV was removed from consideration.

For processing of ribosome profiling data, linker sequences were removed, and samples were demultiplexed using FASTX-clipper and FASTX-barcode-splitter (FASTX-Toolkit v0.0.13). To generate quality and duplication metrics for the entire library, reads were processed with FASTQ-quality-filter with flags -Q33 -q 37 -p 80, then trimmed to remove the 3′ sample barcode (4 nt) and a single base of low quality at the 5′ end (1 nt), and then processed with cd-hit-est (v4.8.1) with flag -c 1 to require 100% identity. For all other analyses, a preliminary alignment of sample barcode- and UMI-trimmed reads to the reference was performed with Bowtie (v1.2.3) and flags -v 1 -k 1 -m 1 to allow a single mismatch; then, the original sequences were retrieved, sample barcodes were trimmed, and quality filtering was performed as described above. At this stage, reads were optionally collapsed on 100% identity of the read+UMI using cd-hit-est as above, and then the UMIs were trimmed. From this step forward, parallel pipelines were used for the collapsed and uncollapsed reads. Reads were finally mapped to the reference using Bowtie as above, with SAMtools (v0.1.19) to generate sorted, indexed BAM files.

For the corresponding RNA-seq data sets, reads were first quality-filtered using PriceSeqFilter (v1.2) with flags -rqf 85 0.98 -rnf 90 and then mapped to the reference using Bowtie2 (v2.2.4) with default parameters. From these BAM files, quantification of reads within the CDS on each strand was performed using Bedtools coverage, and coverage at each nucleotide position was determined using Bedtools genomecov with flag -d. Separate mapping to the reference transcripts plus custom *Culex tarsalis* mitochondrial genome (derived from data from Batson et al. [7]) plus ERCCs validated the strand specificity of library preparation and inclusion of RNA with minimal to no DNA contamination.

Custom scripts were used for further analysis and plotting in Python 3.8, including a combination of plastid (v0.5.1) (63), biopython (v1.77), numpy (v1.19.1), pandas (v1.1.1), scipy (v1.5.2), matplotlib (v3.3.1), and seaborn (v0.10.1). When not otherwise specified, footprint coverage was determined by processing the BAM file via plastid get_count_vectors.py, specifying 27- to 39-nt footprint length and flags –center –nibble 0 –normalize (distributes the count of 1 read across all positions of the read and normalizes by millions of mapped reads in the sample).

For periodicity analysis, footprints were first mapped to the 5′ end using plastid with –fiveprime –normalize and then scaled within each refseq+sample pairing to be comparable across samples. Selecting the CDS region with 25-nt padding from either end, the power spectral density was estimated using Welch’s method performed by scipy.signal.welch with parameters window=‘hann’, nperseg=500, noverlap=250, scaling=‘density’, average=‘median’.

For modeling the impact of nucleotide bias on footprint position, we first developed a footprint generator as follows: choice of 5′ cut site on a given sequence using numpy’s random.default_rng function with probabilities based on the empirical observations of bases found at the positions on either side of the 5′ cut site; then choice of 3′ cut site based on similar choice with base-preference weighting at the 3′ end in addition to weighting by empirical length distribution. This generator was run to produce 10,000 footprints from both the actual and a scrambled CxNV1 RdRp sequence and then was repeated for a total of 10 runs. The coverage of modeled footprints in each scenario was then compared to the observed coverage using Pearson correlation.

For RNA secondary structure analyses, RNAfold (v2.4.0) from the ViennaRNA package was used to calculate minimum free energy along sliding windows of the stated sizes, with a temperature of 28°C (64).

For analyses of footprint context (RNA secondary structure and amino acid features), a window of adjacent nucleotides/amino acids was first selected. For amino acid analysis, this window included 20 residues upstream of the codon at 2/3 of the distance from the 5′ end of the footprint, roughly corresponding to residues likely to be within the ribosome exit tunnel (28). Features of the sequence within the selected window were then averaged across footprint positions. For analysis probing RNA secondary structure at the edge of footprints, the “local MFE peak” was calculated as (MFE_x_ – average[MFE_(x–win/2)_, MFE_(x+win/2)_]), where MFE_x_ is the MFE at position x and win is the window size, and then compared to the numpy gradient function of the footprint profiles (“footprint boundaries,” emphasizing the plateau edges as steep changes in the coverage).

### Polysome profiling.

CT cell lysates were prepared and separated on 10 to 50% mass/vol sucrose gradients as for ribosome profiling above, except without any RNase digestion step. In addition, the lysate was separated into a control condition or treated with 30 mM EDTA and then incubated on ice for 5 min prior to loading onto sucrose gradients. Fractions were then collected using the Biocomp Gradient Station for the full volume that could be sampled, which excludes the small amount of residual volume of densest sucrose and any pelleted material.

An aliquot of each fraction was then mixed 1:1 with 2× DNA/RNA Shield (Zymo Research) spiked with *in vitro* transcribed RNA (generated by T7 transcription off a Firefly luciferase [Fluc]-containing plasmid). RNA extraction was performed in 96-well format on a Bravo automated liquid handling platform (Agilent) using the Quick-DNA/RNA viral MagBead kit (Zymo Research) with proteinase K and DNase steps. RT-qPCR was then performed in 384-well format with a Luna universal probe one-step RT-qPCR kit (NEB), assaying Fluc (primers oHR711/712, FAM probe oHR713), GAPDH (oHR720/721/722), CxNV1 RdRp (oHR729/730/731), and CxNV1 Robin (oHR738/739/740), in triplicate wells for each RNA-target gene combination (Table S9).

Standard analysis was performed on quantification cycle (*C_q_*) values using empirical amplification efficiencies for each primer/probe set and the delta-delta threshold cycle (*C_T_*) method to normalize by Fluc. For each target gene, the relative RNA quantities in each fraction were then normalized across all fractions sampled in the gradient. For each lysate/treatment combination, 2 to 3 technical replicates were run on separate sucrose gradients and subsequent steps. A total of 2 biological replicates of cell lysates were collected for the non-EDTA condition.

### Data availability.

The complete genome sequence of CxNV1 is available at GenBank under accession numbers MW226855 and MW226856. Raw NGS data have been deposited in the NCBI Sequence Read Archive (SRA) under BioProject number PRJNA675022. Raw mass spectrometry data have been deposited at ProteoSAFe (massive.ucsd.edu) under accession number MSV000086532. Supplementary materials are available at https://doi.org/10.7272/Q6GX48SV.
